# A prediction model of contrast-associated acute kidney injury in patients with hypoalbuminemia undergoing coronary angiography

**DOI:** 10.1186/s12872-020-01689-6

**Published:** 2020-08-31

**Authors:** Liwei Liu, Jin Liu, Li Lei, Bo Wang, Guoli Sun, Zhaodong Guo, Yibo He, Feier Song, Zhubin Lun, Bowen Liu, Guanzhong Chen, Shiqun Chen, Yongquan Yang, Yong Liu, Jiyan Chen

**Affiliations:** 1 The Second School of Clinical Medicine, Southern Medical University, Guangzhou, 510515 Guangdong China; 2 Department of Cardiology, Guangdong Provincial Key Laboratory of Coronary Heart Disease Prevention, Guangdong Cardiovascular Institute, Guangdong Provincial People’s Hospital, Guangdong Academy of Medical Sciences, Guangzhou, 510080 China; 3 Department of Emergency and Critical Care Medicine, Guangdong Provincial People’s Hospital and Guangdong Academy of Medical Sciences, Guangzhou, 510080 People’s Republic of China; 4 Department of Cardiology, Dongguan People’s Hospital, Dongguan, 523059 China; 5 Guangdong Provincial People’s Hospital, School of Medicine, South China University of Technology, Guangzhou, 510100 China

**Keywords:** Contrast-associated kidney injury, Hypoalbuminemia, Nomogram, Coronary angiography, Percutaneous coronary intervention

## Abstract

**Background:**

Risk stratification is recommended as the key step to prevent contrast-associated acute kidney injury (CA-AKI) among at-risk patients following coronary angiography (CAG) and/or percutaneous coronary intervention (PCI). Patients with hypoalbuminemia are prone to CA-AKI and do not have their own risk stratification tool. Therefore, this study developed and validated a new model for predicting CA-AKI among hypoalbuminemia patients CAG/PCI.

**Methods:**

1272 patients with hypoalbuminemia receiving CAG/PCI were enrolled and randomly allocated (2:1 ratio) into the development cohort (*n* = 848) and the validation cohort (*n* = 424). CA-AKI was defined as an increase of ≥0.3 mg/dL or 50% in serum creatinine (SCr) compared to baseline in the 48 to 72 h after CAG/PCI. A prediction model was established with independent predictors according to stepwise logistic regression, showing as a nomogram. The discrimination of the new model was evaluated by the area under the curve (AUC) and was compared to the classic Mehran CA-AKI model. The Hosmer-Lemeshow test was conducted to assess the calibration of our model.

**Results:**

Overall, 8.4% (71/848) patients of the development group and 11.2% (48/424) patients of the validation group experienced CA-AKI. A new nomogram included estimated glomerular filtration rate (eGFR), serum albumin (ALB), age and the use of intra-aortic balloon pump (IABP); showed better predictive ability than the Mehran score (C-index 0.756 vs. 0.693, *p* = 0.02); and had good calibration (Hosmer–Lemeshow test *p* = 0.187).

**Conclusions:**

We developed a simple model for predicting CA-AKI among patients with hypoalbuminemia undergoing CAG/PCI, but our findings need validating externally.

**Trial registration:**

http://www.ClinicalTrials.gov NCT01400295, retrospectively registered 21 July 2011.

## Background

Contrast-associated acute kidney injury (CA-AKI) is one of the major complications that occur after contrast exposure [1], leading to higher mortality and worse clinical outcomes [2]. The guidelines recommend that the risk of CA-AKI should be assessed among patients receiving contrast exposure [3].

Hypoalbuminemia is a frequent problem in patients with cardiovascular disease, with a prevalence ranging from 10 to 40% in coronary artery disease patients [4–7]. A previous study has reported that hypoalbuminemia is an independent risk factor for CA-AKI [8]. Elderly age, inflammation and comorbidities, considered as main factors in the occurrence of hypoalbuminemia [9], are also the important mechanism for developing CA-AKI. Moreover, the development of AKI is associated with worse survival than the absence of AKI in patients with baseline hypoalbuminemia [10].

Although patients with baseline hypoalbuminemia are more vulnerable to CA-AKI, there is no prediction model to identify such patients. A recent review concluded that only a few published models are available for routine clinical use [11]. Thus, this study intended to establish a new predicting model to assess the risk of CA-AKI among patients with hypoalbuminemia following CAG or PCI.

## Methods

### Patients

From January 2010 to October 2012, this prospective study reviewed patients aged ≥18 years with baseline hypoalbuminemia [hypoalbuminemia was defined as serum albumin less than 3.5 g/L (< 35 mg/dl)] who were undergoing CAG or PCI and who were included in an observational cohort (PREdictive Value of COntrast voluMe to creatinine Clearance Ratio, PRECOMIN, NCT01400295) in Guangdong Provincial People’s Hospital [12, 13]. The criteria of exclusion included lactation, intravascular injection of contrast agents within the 7 days before or 3 days after operation, no use of isotonic saline for hydration, no use of low-osmolarity contrast medium, cardio surgery or endovascular repair therapy, end-stage kidney disease or renal replacement therapy, malignancy, pregnancy, missing data of perioperative creatinine [12]. All patients of our cohort signed informed consent.

Finally, 1272 patients with baseline hypoalbuminemia(< 35 mg/dl) were enrolled in the analysis. All eligible patients were randomly divided (2:1 ratio) into 2 group: development cohort (*n* = 848) and validation cohort (*n* = 424). During the median follow-up time of 7.7 (6.8;8.8) years, the event(all-cause mortality) was recorded by experienced nurses via telephone visits.

### Coronary angiography and percutaneous coronary intervention

Patients suspected of or diagnosed of coronary artery disease were undergoing CAG/PCI. During the operation, standard guidewires, catheters, and stents and the dose of contrast were used and determined by the interventional cardiologist. All procedures were performed according to the guidelines of the American Heart Association/American College of Cardiology Foundation [14].

Based on the guildline [15], each patient received intravenous hydration of isotonic saline with a rate of 1 mL/kg per hour for at least 2 to 12 h before and 6 to 24 h after the CAG/PCI, while 0.5 mL/kg per hour in cases of severe congestive heart failure or left ventricular ejection fraction (LVEF) < 40%.

### Study endpoint and definitions

The endpoint in this study was CA-AKI defined as an increase of ≥0.3 mg/dL or 50% in serum creatinine (SCr) compared to baseline in the 48 to 72 h after CAG/PCI [16]. The definition of CKD was as an estimated glomerular filtration rate (eGFR) < 60 mL/min/1.73 m^2^, which was calculated by the Modification of Diet in Renal Disease (MDRD) formula. The definitions of hypotension, diabetes, IABP, and CHF were the same as those used in the Mehran score [17]. Serum albumin (ALB) was analyzed by an automatic biochemical analyzer *(*Beckman Coulter AU5800, Ireland*).* The Mehran scores included eight variables (age>75 years, hypotension, IABP, CKD, CHF, diabetes, anemia and contrast volume) [17], and the score of each patient was calculated by trained clinicians. Experienced nurses recorded follow-up of mortality via telephone visits every year after enrollment till April 2019.

### Statistical analysis

Comparisons between normally distributed continuous variables were performed using t-tests, expressed as the mean ± SD. While nonnormally distributed variables were examined via the Wilcoxon rank-sum test, presented as the median ± interquartile. The Pearson χ2 or Fisher exact tests were used for categorical data, shown as percentages. Kaplan-Meier curves were plotted to explore the relationship between CA-AKI and all-cause death among patients with or without hypoalbuminemia. The impact of CA-AKI on long-term mortality among patients with or without hypoalbuminemia was assessed using multivariate Cox regression [18, 19].

The associations between CA-AKI and variables in the development cohort were assessed by univariable logistic analysis. Collinearity between variables was evaluated. Variables were included in the multivariable analysis using a cut-off of *P* < 0.05 in univariate logistics regression. Backward stepwise regression was conducted to select factors and develop our final model, showing as a nomogram. The regression coefficient of each variable in the model was transformed into a 0 to 100 point scale. The total points were calculated by adding points of each variable and then turned into predicted probabilities. A receiver operating characteristic (ROC) curve and AUC were used to assess the discrimination of the nomogram in both the development and validation cohorts compared to the Mehran score. Internal validation was analyzed using 1000 bootstrap samples [13, 20]. The Hosmer-Lemeshow test and a calibration curve were used to assess the calibration of our model. Variables with missing values > 15% were not considered candidates. All statistical analyses were analyzed in SPSS software (ver. 25.0) and R software (ver. 3.6.2).

## Results

### Baseline clinical characteristics

During this study period, 1272 consecutive patients who had baseline hypoalbuminemia were enrolled. The incidence of CA-AKI was 9.36% (119/1272). Patients with baseline hypoalbuminemia were elderly (mean age: 66.00 ± 10.63 years) and having many comorbidities, such as CKD (28%), acute myocardial infarction (AMI) (45.8%), anemia (41.8%). The incidence of CA-AKI in the development cohort was 8.4% (71/848), and in the validation cohort was 11.2% (48/424). No significant difference was identified between these two cohorts, except for in hydration volume (Table 1). More contrast volume was used in patients undergoing PCI, but no statistical difference was shown in the prevalence of CA-AKI (Supplementary Table 1).

**Table 1:** Baseline characteristics of the development cohort and validation cohort

	Development (*n* = 844)	Validation (*n* = 428)	p
Age, y	66.02 (10.44)	65.96 (11.02)	0.921
Female, n (%)	204 (24.2)	82 (19.2)	0.051
Weight, n	63.48 (10.10)	62.62 (10.49)	0.361
SBP, mmHg	127.66 (20.48)	128.85 (20.78)	0.329
DBP, mmHg	74.30 (12.08)	74.92 (12.31)	0.385
HR, bpm	75.61 (13.73)	75.60 (14.41)	0.989
**Medical history**			
CKD, n (%)	251 (29.7)	105 (24.5)	0.059
AMI, n (%)	378 (45.1)	202 (47.3)	0.495
Hypertension, n (%)	494 (58.5)	250 (58.5)	1
Pre-hypotension, n (%)	13 (1.5)	9 (2.1)	0.621
Hyperlipidemia, n (%)	99 (11.7)	40 (9.3)	0.233
Anemia, n (%)	346 (41.6)	179 (42.2)	0.878
DM, n (%)	219 (25.9)	96 (22.5)	0.2
CHF, n (%)	198 (23.6)	87 (20.4)	0.227
LVEF, n	56.28 (12.98)	55.39 (12.16)	0.262
NYHA	1.92 (0.76)	1.87 (0.67)	0.317
**Laboratory examination**			
eGFR, mL/min/1.73 m^2^	72.30 (22.57)	73.42 (21.68)	0.395
Scr, μmol/L	103.03 (59.22)	100.22 (40.16)	0.379
ALB, g/l	31.59 (2.72)	31.60 (2.74)	0.96
Lpa, mg/dl	34.55 (36.49)	34.10 (38.28)	0.849
BUN, mg/dl	5.61 (3.03)	5.67 (3.02)	0.75
Na, mmol/L	138.82 (3.40)	138.42 (3.43)	0.097
K, mmol/L	3.74 (0.46)	3.72 (0.43)	0.547
**Medications**			
Metformin, n (%)	31 (3.7)	16 (3.7)	1
ACEI/ARB, n (%)	735 (87.1)	388 (90.7)	0.075
Diuretics, n (%)	187 (22.2)	97 (22.6)	0.603
Beta blocker, n (%)	678 (80.3)	362 (84.6)	0.076
**Procedure**			
Contrast volume, mL	133.99 (66.22)	131.97 (63.40)	0.603
IABP, n (%)	43 (5.1)	18 (4.2)	0.574
Hydration volume, mL	874.12 (516.30)	816.60 (418.54)	0.047
**Other**			
CA-AKI, n (%)	71 (8.4)	48 (11.2)	0.129
Mehran score	5.75 (4.74)	5.25 (4.61)	0.075

Abbreviations: *CA-AKI* Contrast-associated acute kidney injury, *SBP* Systolic blood pressure, *DBP* Diastolic blood pressure, *HR* Heart rate, *CKD* Chronic kidney disease, *AMI* Acute myocardial infarction, *DM* Diabetes mellitus, *CHF* Congestive heart failure, *LVEF* Left ventricular ejection fraction, *NYHA* NYHA classification grading of cardiac function, *eGFR* Estimated glomerular filtration rate, *ALB* Serum albumin, *Lpa* Lipoprotein a, *BUN* Blood urea nitrogen, *IABP* Intra-aortic balloon pump, *Scr* Serum creatinine

Patients with CA-AKI tended to have lower eGFR, LVEF, ALB and higher age, heart rate (HR), and SCR in the development cohort (Supplementary Table 2). At baseline, CA-AKI patients had more complications, such as CKD, CHF, AMI, hypertension, and anemia.

### Development and validation

The results of univariate logistic analysis about associations with CA-AKI are shown in Table 2. After multivariate stepwise selection, IABP (OR: 8.267, 95% CI: 4.007–16.978), eGFR (OR: 0.982, 95% CI: 0.970–0.994) and ALB (OR: 0.875, 95% CI: 0.801–0.955), age (OR: 1.036, 95% CI: 1.006–1.007) were included in the final model for predicting CA-AKI (Table 3).

**Table 2:** Univariate logistic regression

	OR	95% CI		P
ALB	0.817	0.756–0.883		< 0.001
Contrast volume	1.003	0.999–1.006		0.136
DM	1.514	0.889–2.517		0.117
Pre-hypotension	3.459	0.762–11.633		0.064
PCI	1.387	0.804–2.512		0.257
eGFR	0.968	0.957–0.979		< 0.001
Scr	1.006	1.003–1.010		< 0.001
Age	1.058	1.030–1.088		< 0.001
AMI	2.113	1.290–3.520		0.003
NYHA	2.290	1.587–3.308		< 0.001
CKD	3.664	2.237–6.066		< 0.001
Hypertension	2.404	1.397–4.342		0.002
IABP	12.788	6.558–24.868		< 0.001
CHF	3.848	2.34–6.338		< 0.001
LVEF	0.966	0.949–0.984		< 0.001
HR	1.018	1.005–1.031		0.005
Anemia	1.802	1.106–2.956		< 0.001
BUN	1.131	1.067–1.197		< 0.001
Diuretics	1.658	1.084–2.465		0.015
Beta-blocker	0.510	0.302–0.885		0.014
Hydration volume	1.001	1.000–1.001		< 0.001

Abbreviations: *CA-AKI* Contrast-associated acute kidney injury, *eGFR* Estimated glomerular filtration rate, *AMI* Acute myocardial infarction, *IABP* Intra-aortic balloon pump, *CHF* Congestive heart failure, *LVEF* Left ventricular ejection fraction, *HR* Heart rate, *ALB* Serum albumin, *BUN* Blood urea nitrogen; infarction, *Scr* Serum creatinine, *PCI* Percutaneous coronary intervention

**Table 3:** Multivariate logistic regression

	OR	95% CI	p
eGFR	0.982	0.970–0.994	0.004
ALB	0.875	0.801–0.955	0.002
IABP	8.267	4.007–16.978	< 0.001
Age	1.036	1.006–1.067	0.018

Abbreviations: *eGFR* Estimated glomerular filtration rate, *ALB* Serum albumin, *IABP* Intra-aortic balloon pump

**Fig. 1:**
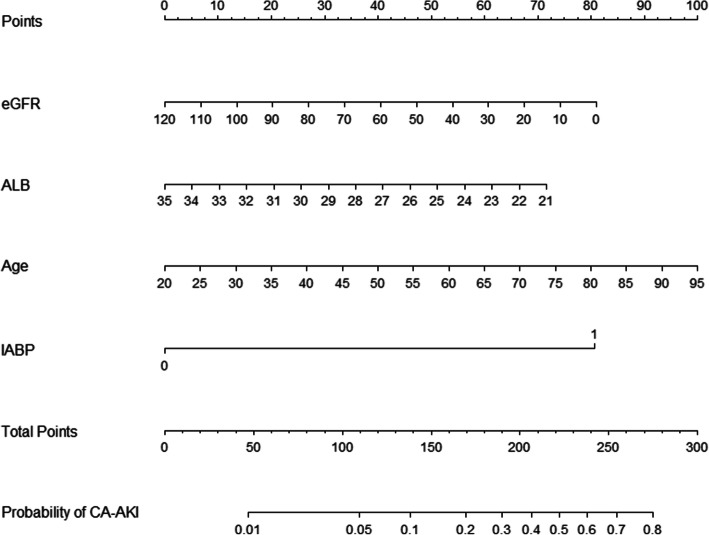
The nomogram of the new model for CA-AKI

**Fig. 2:**
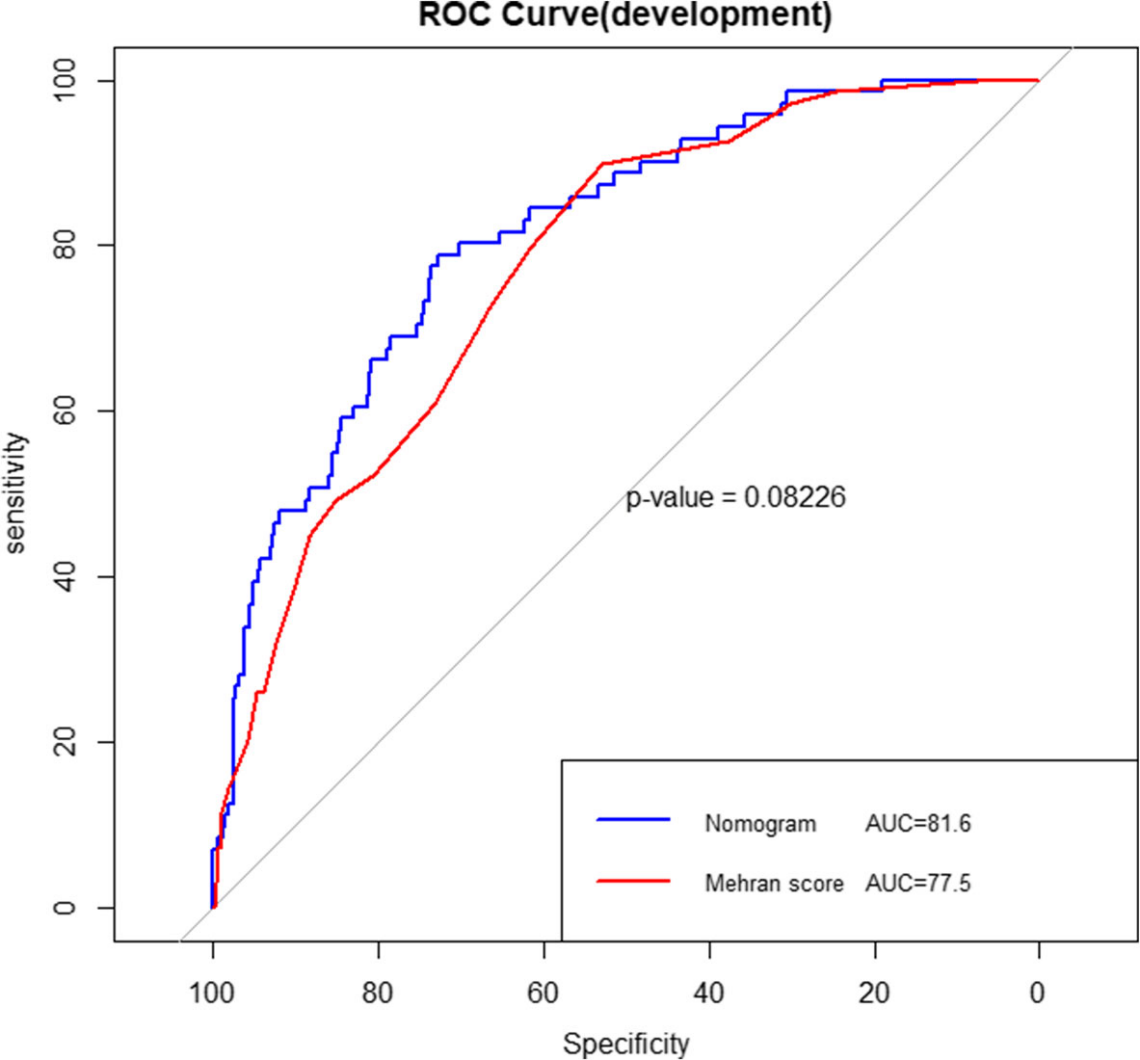
The ROC curves of the two models for CA-AKI in the development cohort

**Fig. 3:**
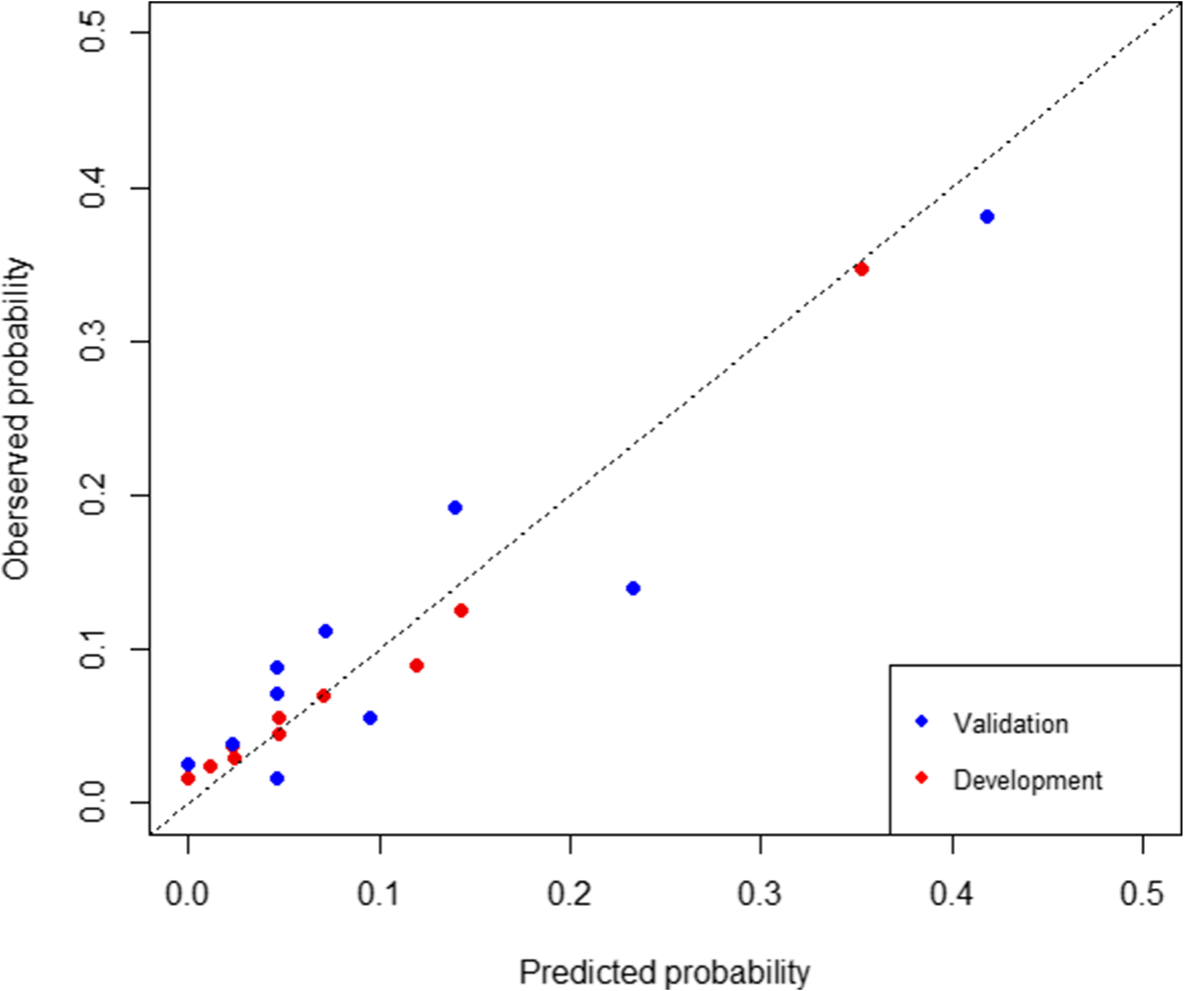
The calibration curve of the new model in the development cohort and validation cohort

Based on these independent risk factors, a simple nomogram was formed (Fig. 1). In the development cohort, the nomogram displayed good discriminative power of predicting the CA-AKI (AUC 0.816, 95% CI: 0.763–0.862; bootstrap corrected C-index 0.802). Compared to the Mehran score, this nomogram had good discrimination (AUC 0.816 VS 0.775, shown in Fig. 2). Besides, calibration plots graphically presented great agreement regarding the development of CA-AKI between the risk assessment and the observed frequency (Fig. 3). The Hosmer–Lemeshow statistic suggested a good fit (χ^2^ = 3.65, *P* = 0.887) in the development cohort.

**Fig. 4:**
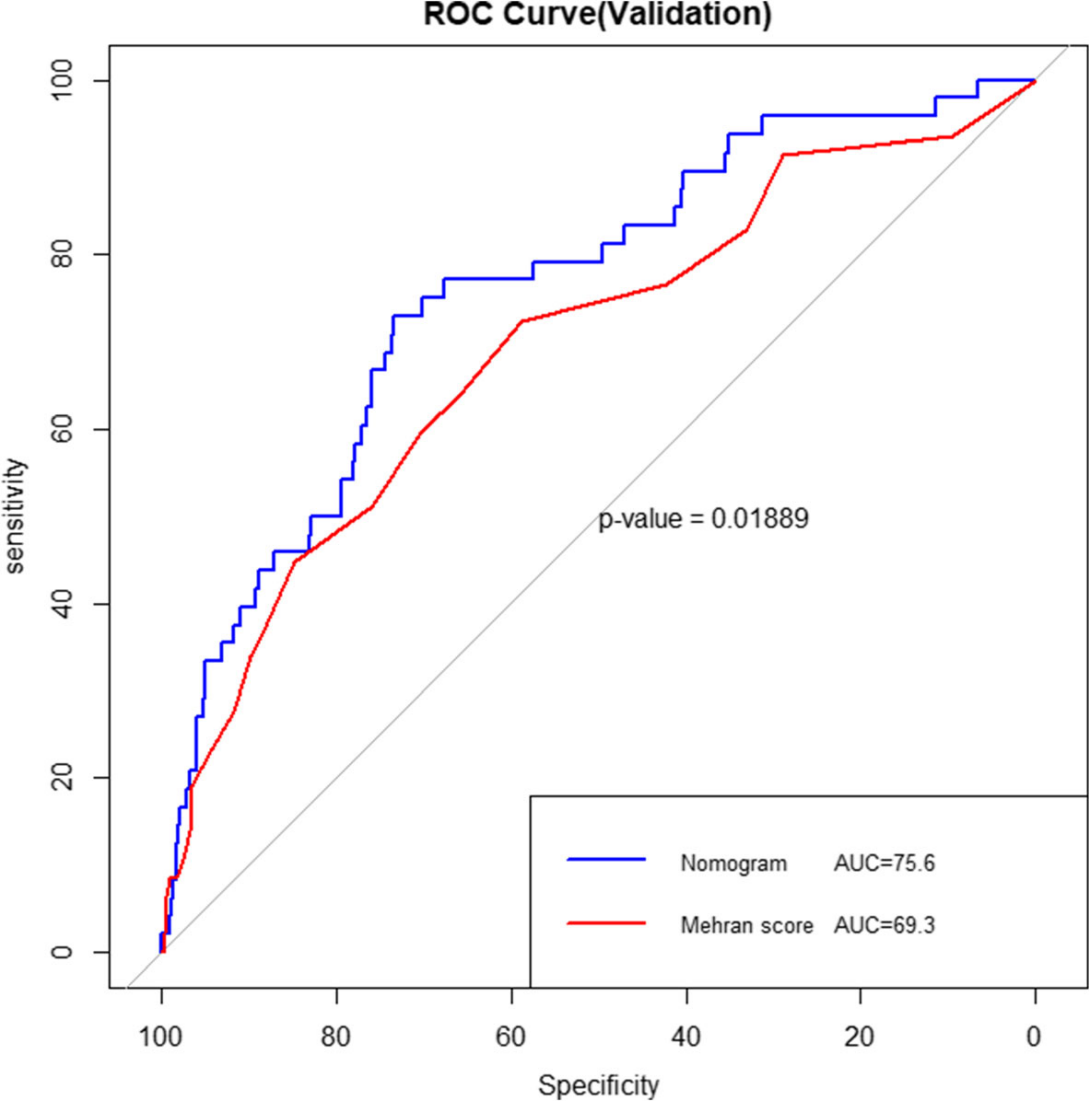
The ROC curves of the two models for CA-AKI in the validation cohort

In the validation group, the new nomogram demonstrated a C-index of 0.756 (95% CI 0.685–0.832), showing better discriminative power than Mehran score (AUC: 0.693, 95% CI 0.608–0.779) among patients (*P* = 0.02, Fig. 4). Besides, our model had a good performance of calibration in the validation cohort (χ^2^ = 11.27, *P* = 0.187, Fig. 3). The clinical usefulness of the new nomogram was better than Mehran score in the development and validation cohort (Supplementary figure 1A and B).

### Follow-up of clinical outcomes

**Fig. 5:**
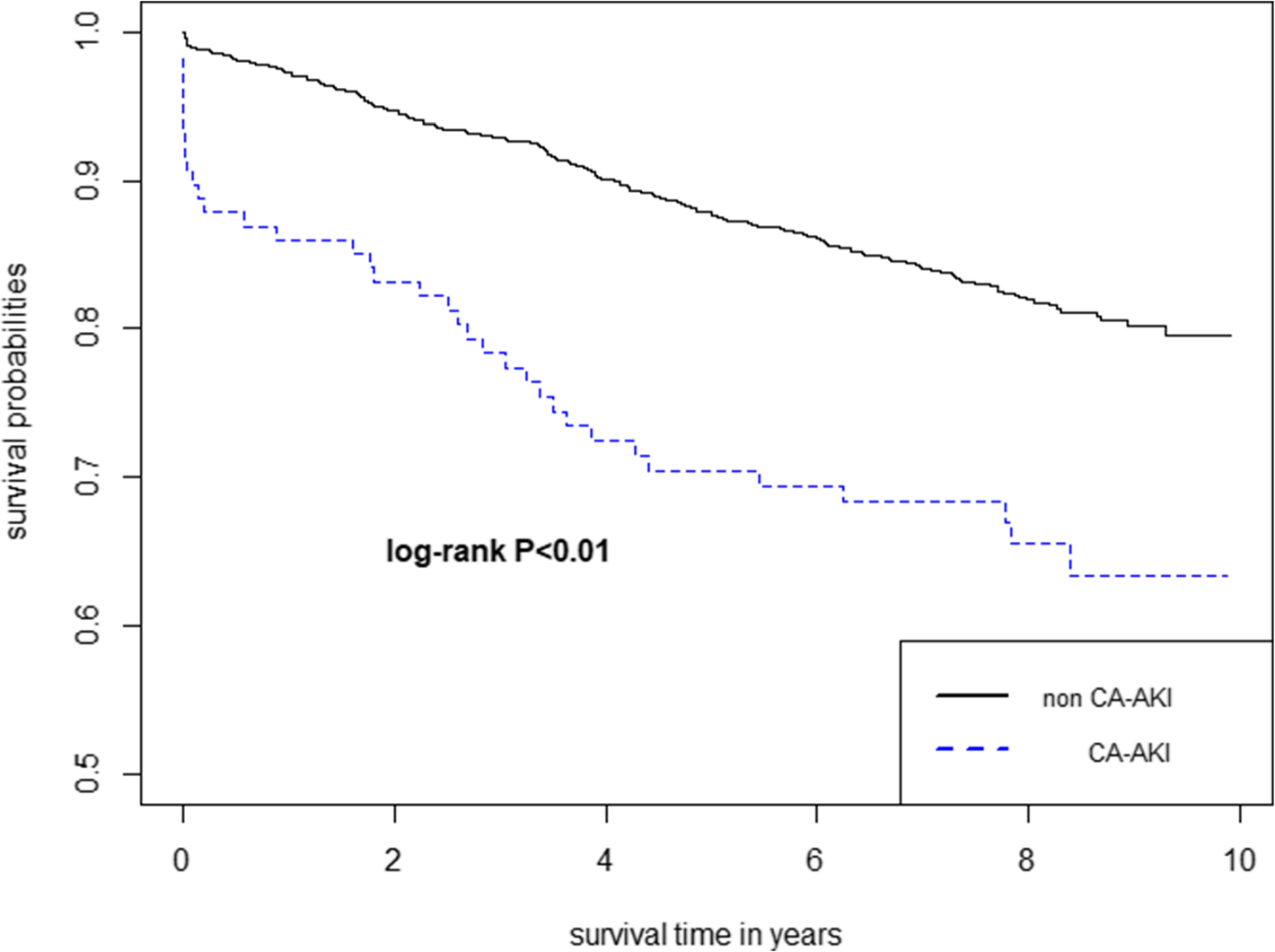
The Kaplan–Meier curve to estimates the impact of CA-AKI on long-term mortality in patients with hypoalbuminemia

We divided patients into 2 groups according to whether they developed CA-AKI. During the follow-up time, the rates of mortality were 30.2 and 17.1% in the CA-AKI group and non-CA-AKI group, respectively. According to the log-rank analysis (Fig. 5), the occurrence of CA-AKI presented higher long-term mortality (*P* < 0.01) in patients with hypoalbuminemia. By multivariate Cox regression analysis, CA-AKI was an independent prognostic risk factor in patients with hypoalbuminemia (Hazard ratio:1.525, 95% CI 1.03–2.25) after adjusting for diabetes, age, gender, hypertension, IABP and eGFR (Supplementary Table 3). Similarly, among patients with normal albumin levels, the occurrence of CA-AKI presented worse prognosis (Log-rank analysis *P* < 0.01, Supplementary Figure 2). CA-AKI was independently associated with the long-term mortality (Hazard ratio:1.489, 95% CI 1.013–2.187) in a prognostic model (Supplementary Table 3).

## Discussion

Our study was the first to develop a CA-AKI risk stratification model in a population with hypoalbuminemia. We established a simple nomogram that included four powerful predictors for clinical use (IABP, ALB, eGFR, age). Compared to the classical Mehran score, the new model had good discrimination and calibration in predicting CA-AKI.

Hypoalbuminemia is associated with several pathological conditions. Yu et al. [10] showed that hypoalbuminemia often was observed in elderly patients and in patients having many comorbidities. Similarly, in our cohort, most patients were older and accompanying CKD, AMI, anemia and CHF, which may lead to the status of low serum albumin level. The overall incidence of CA-AKI was 9.36% in this study, which was relatively high but similar to the high-risk population, such as patients with CKD or AMI [21, 22]. Several studies have demonstrated that hypoalbuminemia is an independent risk factor for CA-AKI [8] and is closely related to poor prognosis in coronary heart disease [23]. Yu et al. [10] reported that patients with hypoalbuminemia had a high incidence of AKI in the hospital. Furthermore, in our study, the development of AKI in patients was associated with a long-term mortality according to the results of the 7-year follow-up, which was similar to a previous study [10]. Due to the poor prognosis of CA-AKI in patients with known hypoalbuminemia, it is necessary for clinicians to early identify the high-risk individual of CA-AKI, leading to prompt management and intervention.

Risk assessment in high-risk groups is a primary aim and important for the prevention of CA-AKI, so a large number of models have been proposed [11]. Although the classic Mehran score or other models had good predictive efficiency, most models included 5–8 variables, and some factors required the subjective judgment of clinicians. In recent years, predictive models have been established for different populations at high risk of CA-AKI, such as patients with CKD [24], AMI [25] or diabetes mellitus [26]. However, there was no predictive model for patients with hypoalbuminemia. Because of the high incidence of CA-AKI in patients with hypoalbuminemia, it is essential to develop a simple risk score for these patients undergoing CAG/PCI.

For predicting CA-AKI, the currently available models seldom included albumin [11] because serum albumin was a novel laboratory risk factor. Murat et al. [8] suggested that albumin is an independent and good predictor of CA-AKI among patients with ACS undergoing PCI. Low serum albumin concentrations reflect the inflammatory state of the body, which may lead to CA-AKI [9].

The use of IABP was the strongest predictor of CA-AKI in the present model. Previous studies reported that IABP reflected the unstable hemodynamics and was independently associated with CA-AKI [24, 27]. Bartholomew et al. [28] firstly showed a close relationship between the use of IABP and CA-AKI, and then Mehran et al. [17] first included IABP in a model of CA-AKI prediction. Perioperative hemodynamic abnormalities may result in ischemia-reperfusion injury, which could lead to a potential impact on the AKI.

Currently, age was an independent predicting variable of the occurrence of CA-AKI in the known risk model [17, 29]. This independent predictive ability may be related to a degenerative change in the structure and function of kidneys with increasing age. Baseline eGFR was also a common risk factor for CA-AKI following CAG [30]. eGFR represents worse kidney function and a higher risk of acute kidney injury [31].

Diabetes and contrast volume were not included in our nomogram, although these variables were included in previous models [32]. In our study, diabetes and contrast volume were not independent risk factors for CA-AKI based on statistical analysis. Similar to our finding, Sabeti et al. [33] showed that diabetes mellitus was not an independent risk factor for CA-AKI. A recent review concluded that diabetes is not independently associated with the risk of developing CA-AKI and only increases susceptibility in patients with underlying kidney dysfunction [34]. Current studies hold that the comorbidities and hemodynamic instability were more related to the occurrence of AKI after contrast exposure rather than high volume of contrast [35, 36]. In our cohort, the association between contrast volume and CA-AKI showed no statistically significant difference. One of possible reasons for this result is that hypoalbuminemia patients tend to have more comorbidities and unstable hemodynamics [10]. As for anemia and LVEF, these factors were associated with CA-AKI in univariable, but not in multivariable analysis, which was similar to other studies [11, 37]. Our results suggested that the impact of anemia on CA-AKI may be potentially affected by confounding factors, like renal dysfunction or age. Similarly, Gao et al. showed the LVEF was not independently related to CA-AKI when adjusting for age or IABP [38]. The KDIGO guidelines [39] suggest that eGFR should be used instead of Scr to assess baseline renal function in patients before the administration of contrast medium because Scr is also affected by age, gender, and race. Hydration has been considered an effective treatment for CA-AKI, but the actual effect of this strategy is still controversial. Perioperative hydration can expand plasma volume by inhibiting the renin-angiotensin-aldosterone system, reducing the concentration of contrast media in the tubule lumen and vasa recta, and counteraction of medullary vasoconstriction activation [40].. It is still the cornerstone of CA-AKI prevention. But, the rate and duration of hydration remain inconsistent. Meanwhile, the AMACING trial challenged the tenet that intravenous fluids are effective. The incidence of CA-AKI between the hydration group and the non-hydration group (2.7% vs 2.6%) showed no statistical significant difference [41]. In our study, the volume of hydration was not significantly different in multivariate regression, so this variable was not included in our model. By using our nomogram, clinicians can identify high-risk patients with CA-AKI early and treat them in a timely manner.

## Limitations

Firstly, the data of our study were from a single-center hospital. But this cohort was a large database about CA-AKI in China focusing on patients with hypoalbuminemia.

Secondly, our model is not as effective as other models, but it has fewer objective variables and higher clinical operability. Importantly, our model has good predictive and evaluative effectiveness in hypoalbuminemia populations.

Thirdly, the nomogram established has not been externally verified. However, we randomly divided all the patients into a development group and a validation group according to a 2:1 ratio, which showed that our model had good stability.

Finally, our study lacked data of creatinine after 3 days because several patients were discharged on the third day after CAG/PCI. This may affect the incidence of CA-AKI.

## Supplementary information

**Additional file 1: Table S1.** Baseline characteristics of non-PCI group and PCI group in total cohort. **Table S2.** Baseline characteristics of CA-AKI group and non CA-AKI group in developing cohort. **Table S3.** Multivariate Cox regression analysis of risk factors for long-term mortality. **Table S4.** Multivariate logistic regression of variates with statistically significant difference in univariate analysis.

**Additional file 2: Figure S1.** Decision curve analysis for the development cohort (A) and the validation cohort (B).

**Additional file 3: Figure S2.** Association between CA-AKI and long-term mortality in patients without hypoalbuminemia.

## Data Availability

Data relevant to this study are available from the corresponding authors upon reasonable request.

## Abbreviations

CA-AKI: Contrast-associated acute kidney injury
DBP: Diastolic blood pressure
SBP: Systolic blood pressure
CKD: Chronic kidney disease
HR: Heart rate
AMI: Acute myocardial infarction
DM: Diabetes mellitus
NYHA: NYHA classification grading of cardiac function
eGFR: Estimated glomerular filtration rate
CHF: Congestive heart failure
LVEF: Left ventricular ejection fraction
IABP: Intra-aortic balloon pump
ALB: Serum albumin
BUN: Blood urea nitrogen
Scr: Serum creatinine
