# Supplementary material for: A prediction model of contrast-associated acute kidney injury in patients with hypoalbuminemia undergoing coronary angiography
Source: BMC Cardiovasc Disord. 2020 Aug 31;20:399. doi: 10.1186/s12872-020-01689-6 (PMC7460778; doi:10.1186/s12872-020-01689-6)
Supplement: Supplementary file 1 — Additional file 1: Table S1. Baseline characteristics of non-PCI group and PCI group in total cohort. Table S2. Baseline characteristics of CA-AKI group and non CA-AKI group in developing cohort. Table S3. Multivariate Cox regression analysis of risk factors for long-term mortality. Table S4. Multivariate logistic regression of variates with statistically significant difference in univariate analysis. [file 12872_2020_1689_MOESM1_ESM.docx]

**Supplementary table 1.** Baseline characteristics of non-PCI group and PCI group in total cohort

|  | non-PCI | PCI | p |
| --- | --- | --- | --- |
|  | n=367 | n=905 |  |
| Age, y | 67.66 (9.80) | 65.33 (10.89) | <0.001 |
| Female, n(%) | 108 (29.4) | 178 (19.7) | <0.001 |
| Weight, n | 59.43 (8.79) | 63.34 (10.26) | 0.086 |
| DBP, mmHg | 75.43 (12.90) | 74.14 (11.83) | 0.086 |
| SBP, mmHg | 130.86 (19.63) | 126.93 (20.86) | 0.002 |
| HR, bpm | 75.63 (15.19) | 75.60 (13.44) | 0.976 |
| CKD, n(%) | 103 (28.1) | 253 (28.0) | 1 |
| Hypertension, n(%) | 226 (61.6) | 518 (57.3) | 0.180 |
| Pre-hypotension, n(%) | 9 (2.5) | 13 (1.4) | 0.300 |
| Hyperlipidemia, n(%) | 27 (7.4) | 112 (12.4) | 0.012 |
| Anemia, n(%) | 177 (49.2) | 348 (38.8) | 0.001 |
| DM, n(%) | 87 (23.8) | 228 (25.2) | 0.645 |
| CHF, n(%) | 68 (18.8) | 217 (24.0) | 0.056 |
| LVEF, n | 55.96 (14.67) | 55.98 (11.91) | 0.976 |
| NYHA, | 1.86 (0.77) | 1.94 (0.70) | 0.126 |
| eGFR, mL/min/1.73mm2 | 70.49 (20.79) | 73.56 (22.80) | 0.026 |
| ALB, g/l | 31.95 (2.57) | 31.45 (2.77) | 0.003 |
| Contrast volume, mL | 90.56 (60.14) | 150.60 (59.04) | <0.001 |
| IABP, n(%) | 14 (3.8) | 47 (5.2) | 0.369 |
| Hydration volume, mL | 817.73 (528.56) | 869.22 (467.88) | 0.091 |
| Mehran score | 5.67 (4.62) | 5.55 (4.74) | 0.685 |
| CA-AKI, n(%) | 31 (8.5) | 88 (9.7) | 0.547 |

Abbreviations: ALB: serum albumin; eGFR: estimated glomerular filtration rate; Scr: serum creatinine; IABP: intra-aortic balloon pump; CHF: congestive heart failure; LVEF: left ventricular ejection fraction; HR: heart rate; ALB: serum albumin; CKD: chronic kidney disease; DM: diabetes mellitus; CA-AKI: contrast-associated acute kidney injury

**Supplementary table 2.** Baseline characteristics of CA-AKI group and non CA-AKI group in developing cohort

|  | CA-AKI(-)  n=777 | CA-AKI(+)  n=71 | p |
| --- | --- | --- | --- |
| Age, y | 65.58(10.36) | 70.89(10.09) | <0.001 |
| Female, n(%) | 185(23.9) | 19(26.8) | 0.698 |
| Weight, n | 63.79(10.15) | 61.45(9.62) | 0.138 |
| SBP, mmHg | 127.32(20.00) | 131.37(25.09) | 0.113 |
| DBP, mmHg | 74.26(12.00) | 74.67(13.02) | 0.786 |
| HR, bpm | 75.31(13.39) | 78.96(16.81) | 0.033 |
| **Medical history** |  |  |  |
| CKD, n(%) | 210(27.2) | 41(57.7) | <0.001 |
| AMI, n(%) | 334(43.5) | 44(62.0) | 0.004 |
| Hypertension, n(%) | 440(56.9) | 54(76.1) | 0.003 |
| Pre-hypotension, n(%) | 10(1.3) | 3(4.3) | 0.145 |
| Hyperlipidemia, n(%) | 92(11.9) | 7(9.9) | 0.75 |
| Anemia, n(%) | 307(40.3) | 39(54.9) | 0.024 |
| DM, n(%) | 195(25.2) | 24(33.8) | 0.151 |
| CHF, n(%) | 162(21.1) | 36(50.7) | <0.001 |
| LVEF, n | 56.83(12.87) | 50.57(12.84) | <0.001 |
| NYHA, | 1.89(0.73) | 2.42(0.94) | <0.001 |
| ACEI/ARB, n(%) | 673(87.1) | 62(87.3) | 1 |
| Diuretic, n(%) | 163(21.1) | 24(23.8) | 0.044 |
| Betablocker, n(%) | 629(81.4) | 49(69.0) | 0.019 |
| **Laboratory examination** |  |  |  |
| eGFR, mL/min/1.73mm2 | 73.70(21.77) | 57.09(25.50) | <0.001 |
| Scrμmol/L | 99.73 (52.69) | 131.80 (63.17) | <0.001 |
| ALB, g/l | 31.74(2.58) | 29.94(3.53) | <0.001 |
| Lpa, mg/dl | 34.38(36.94) | 36.34(31.59) | 0.68 |
| BUN, mg/dl | 5.46(2.84) | 7.22(4.29) | <0.001 |
| Na, mmol/L | 138.82(3.36) | 138.86(3.99) | 0.947 |
| K, mmol/L | 3.74(0.45) | 3.81(0.56) | 0.355 |
| **Medications** |  |  |  |
| Metformin, n(%) | 27(3.5) | 4(5.6) | 0.556 |
| ACEI/ARB,n(%) | 673(87.1) | 62(87.3) | 1 |
| Diuretic, n(%) | 163(21.1) | 24(23.8) | 0.044 |
| Beta-blocker, n(%) | 629(81.4) | 49(69.0) | 0.019 |
| **Procedure** |  |  |  |
| Contrast volume, mL | 132.96(65.78) | 145.21(70.35) | 0.136 |
| IABP, n(%) | 23(3.0) | 20(28.2) | <0.001 |
| Hydration-volume, mL | 850.54 (491.58) | 1127.46 (685.92) | <0.001 |
| PCI, n(%) | 538(69.6) | 54(76.1) | 0.316 |
| **Other** |  |  |  |
| Mehranscore | 5.33(4.44) | 10.39(5.48) | <0.001 |

Abbreviations: CA-AKI: contrast-associated acute kidney injury; SBP: systolic blood pressure; DBP: diastolic blood pressure; HR: heart rate; CKD: chronic kidney disease; AMI: acute myocardial infarction; DM: diabetes mellitus; CHF: congestive heart failure; LVEF: left ventricular ejection fraction; NYHA: NYHA classification grading of cardiac function; eGFR: estimated glomerular filtration rate; ALB: serum albumin; Lpa: lipoprotein a; BUN: blood urea nitrogen; IABP: intra-aortic balloon pump; Scr: serum creatinine; PCI: percutaneous coronary intervention

**Supplementary table 3.** Multivariate Cox regression analysis of risk factors for long-term mortality in patients with hypoalbuminemia or without hypoalbuminemia.

| Variates | Hypoalbuminemia (+)  N=1272 | | | Hypoalbuminemia (-)  N=1650 | | | |
| --- | --- | --- | --- | --- | --- | --- | --- |
|  | HR | 95%CI | P |  | HR | 95%CI | P |
| Age | 1.010 | 0.996-1.023  0.985-0.998  0.65-1.237  1.034-2.249  0.684-1.195  0.79-1.431  0.92-1.007  1.303-3.337 | 0.155 |  | 0.991 | 0.979-1.004 | 0.165 |
| eGFR | 0.991 |  | 0.008 |  | 0.996 | 0.991-1.002 | 0.203 |
| Female | 0.897 |  | 0.508 |  | 0.956 | 0.709-1.288 | 0.768 |
| CA-AKI | 1.525 |  | 0.033 |  | 1.489 | 1.013-2.187 | 0.043 |
| Hypertension | 0.904 |  | 0.478 |  | 1.095 | 0.845-1.419 | 0.491 |
| DM | 1.063 |  | 0.685 |  | 1.080 | 0.808-1.442 | 0.603 |
| ALB | 0.962 |  | 0.095 |  | 0.993 | 0.939-1.050 | 0.805 |
| IABP | 2.085 |  | 0.002 |  | 1.944 | 0.943-4.010 | 0.072 |

Abbreviations: CA-AKI: contrast-associated acute kidney injury; eGFR: estimated glomerular filtration rate; DM: diabetes mellitus; ALB: serum albumin; IABP: intra-aortic balloon pump

**Supplementary table 4** Multivariate logistic regression of variates with statistically significant difference in univariate analysis

|  | OR | 95%CI | P |
| --- | --- | --- | --- |
| ALB | 0.873 | 0.768-0.991 | 0.036 |
| Contrast volume | 1.003 | 0.997-1.008 | 0.327 |
| Pre-hypotension | 4.138 | 0.789-21.718 | 0.093 |
| eGFR | 0.983 | 0.971-0.995 | 0.006 |
| Scr | 1.000 | 0.991-1.008 | 0.943 |
| Age | 1.054 | 1.005-1.105 | 0.030 |
| AMI | 1.179 | 0.515-2.698 | 0.696 |
| NYHA | 1.117 | 0.526-2.375 | 0.773 |
| CKD | 0.957 | 0.256-3.579 | 0.948 |
| Hypertension | 2.128 | 0.876-5.169 | 0.095 |
| IABP | 3.842 | 1.139-12.958 | 0.030 |
| CHF | 1.647 | 0.438-6.190 | 0.460 |
| LVEF | 0.908 | 0.804-1.025 | 0.119 |
| HR | 1.001 | 0.974-1.029 | 0.955 |
| Anemia | 1.600 | 0.733-3.490 | 0.238 |
| BUN | 0.934 | 0.831-1.049 | 0.249 |
| Diuretic | 0.613 | 0.296-1.272 | 0.189 |
| Beta-blocker | 0.694 | 0.286-1.684 | 0.420 |
| Hydration volume | 1.000 | 1.000-1.001 | 0.645 |

Abbreviations: HR: heart rate; CKD: chronic kidney disease; AMI: acute myocardial infarction; CHF: congestive heart failure; LVEF: left ventricular ejection fraction; NYHA: NYHA classification grading of cardiac function; eGFR: estimated glomerular filtration rate; ALB: serum albumin; IABP: intra-aortic balloon pump; Scr: serum creatinine; BUN: blood urea nitrogen;
