# Supplementary figures and images for: A prediction model of contrast-associated acute kidney injury in patients with hypoalbuminemia undergoing coronary angiography
Source: BMC Cardiovasc Disord. 2020 Aug 31;20:399. doi: 10.1186/s12872-020-01689-6 (PMC7460778; doi:10.1186/s12872-020-01689-6)

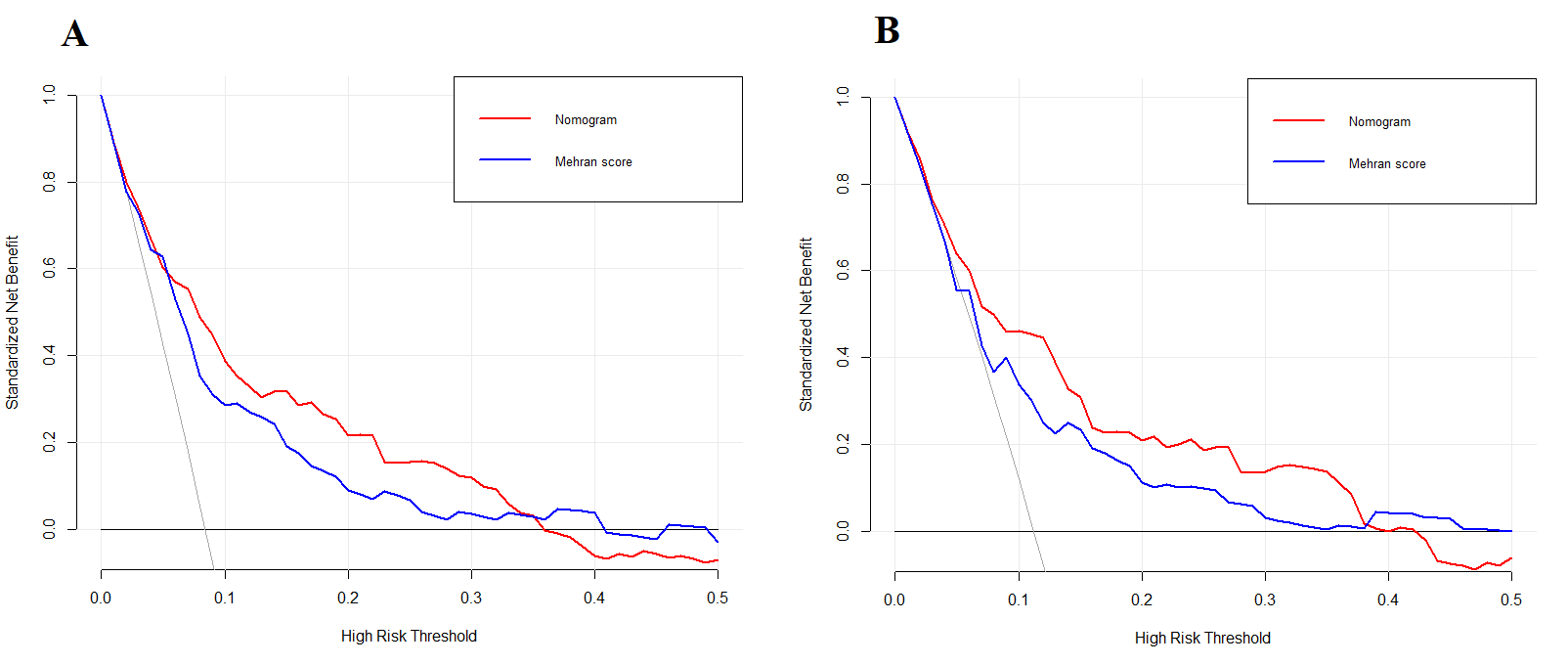

Supplement: Supplementary file 2 — Additional file 2: Figure S1. Decision curve analysis for the development cohort (A) and the validation cohort (B). [file 12872_2020_1689_MOESM2_ESM.tiff]

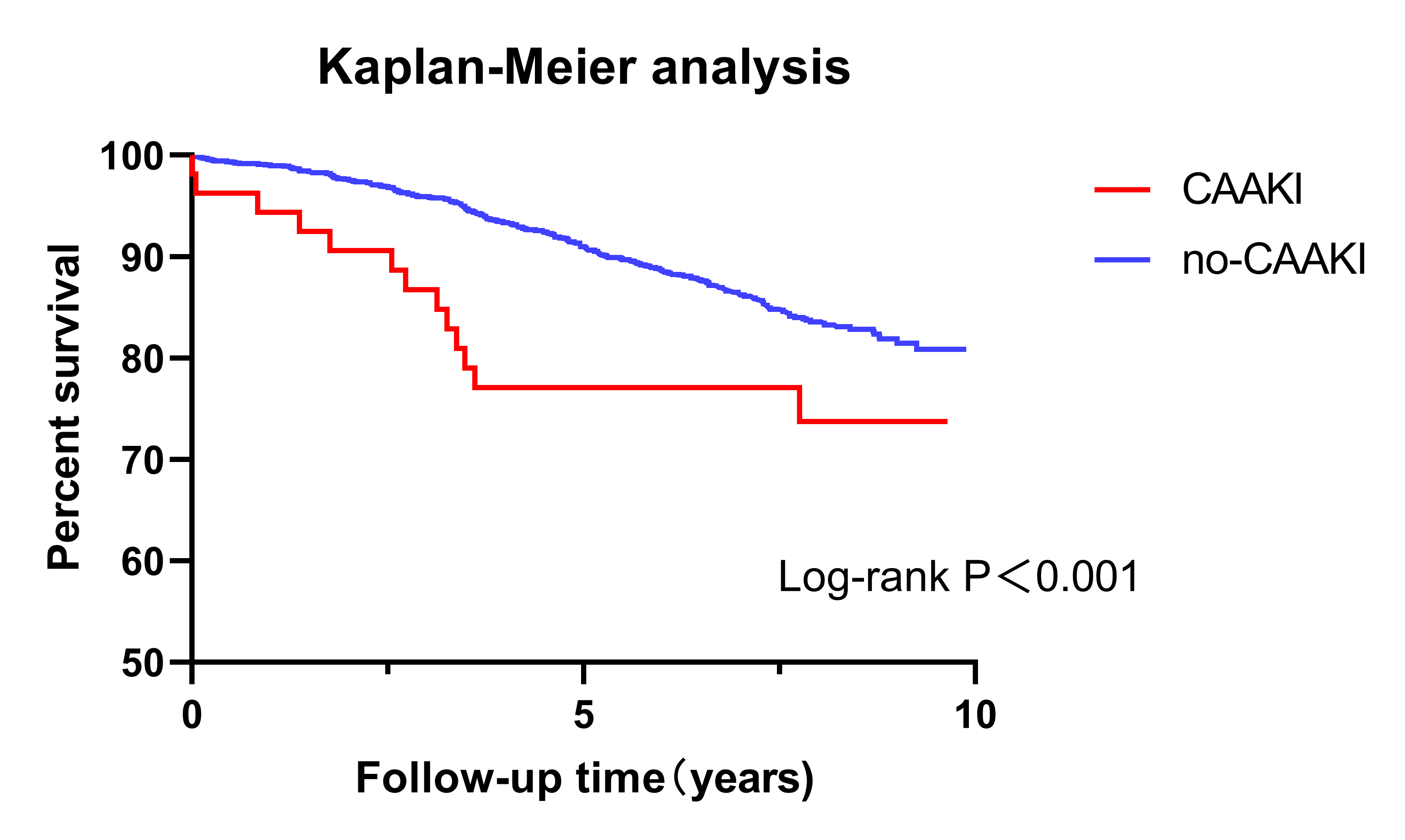

Supplement: Supplementary file 3 — Additional file 3: Figure S2. Association between CA-AKI and long-term mortality in patients without hypoalbuminemia. [file 12872_2020_1689_MOESM3_ESM.tif]
